# Light control of catechin accumulation is mediated by photosynthetic capacity in tea plant (*Camellia sinensis*)

**DOI:** 10.1186/s12870-021-03260-7

**Published:** 2021-10-20

**Authors:** Ping Xiang, Qiufang Zhu, Marat Tukhvatshin, Bosi Cheng, Meng Tan, Jianghong Liu, Xingjian Wang, Jiaxin Huang, Shuilian Gao, Dongyi Lin, Yue Zhang, Liangyu Wu, Jinke Lin

**Affiliations:** 1 College of Horticulture, Fujian Agriculture and Forestry University, Fuzhou, 350002 China; 2 Institute of Photobiological Industry, Fujian Sanan Sino-Science Photobiotech Co., Ltd, Xiamen, 361008 China; 3 Anxi College of Tea Science, Fujian Agriculture and Forestry University, Fuzhou, 350002 China

**Keywords:** Light intensity, Photosynthetic capacity, Catechin biosynthesis, Tea plant

## Abstract

**Background:**

Catechins are crucial in determining the flavour and health benefits of tea, but it remains unclear that how the light intensity regulates catechins biosynthesis. Therefore, we cultivated tea plants in a phytotron to elucidate the response mechanism of catechins biosynthesis to light intensity changes.

**Results:**

In the 250 μmol·m^− 2^·s^− 1^ treatment, the contents of epigallocatechin, epigallocatechin gallate and total catechins were increased by 98.94, 14.5 and 13.0% respectively, compared with those in the 550 μmol·m^− 2^·s^− 1^ treatment. Meanwhile, the photosynthetic capacity was enhanced in the 250 μmol·m^− 2^·s^− 1^ treatment, including the electron transport rate, net photosynthetic rate, transpiration rate and expression of related genes (such as *CspsbA, CspsbB, CspsbC, CspsbD, CsPsbR* and *CsGLK1*). In contrast, the extremely low or high light intensity decreased the catechins accumulation and photosynthetic capacity of the tea plants.

The comprehensive analysis revealed that the response of catechins biosynthesis to the light intensity was mediated by the photosynthetic capacity of the tea plants. Appropriately high light upregulated the expression of genes related to photosynthetic capacity to improve the net photosynthetic rate (Pn), transpiration rate (Tr), and electron transfer rate (ETR), which enhanced the contents of substrates for non-esterified catechins biosynthesis (such as EGC). Meanwhile, these photosynthetic capacity-related genes and gallic acid (GA) biosynthesis-related genes (*CsaroB, CsaroDE1, CsaroDE2* and *CsaroDE3*) co-regulated the response of GA accumulation to light intensity. Eventually, the epigallocatechin gallate content was enhanced by the increased contents of its precursors (EGC and GA) and the upregulation of the *CsSCPL* gene.

**Conclusions:**

In this study, the catechin content and photosynthetic capacity of tea plants increased under appropriately high light intensities (250 μmol·m^− 2^·s^− 1^ and 350 μmol·m^− 2^·s^− 1^) but decreased under extremely low or high light intensities (150 μmol·m^− 2^·s^− 1^ or 550 μmol·m^− 2^·s^− 1^). We found that the control of catechin accumulation by light intensity in tea plants is mediated by the plant photosynthetic capacity. The research provided useful information for improving catechins content and its light-intensity regulation mechanism in tea plant.

**Supplementary Information:**

The online version contains supplementary material available at 10.1186/s12870-021-03260-7.

## Background

Catechins, which are the major secondary metabolites in tea, promote the sensory qualities and health benefits of tea, including cancer prevention and antioxidant activity [1–3], neurodegenerative disease prevention and diabetes prevention [4, 5]. On the basis of whether the galloyl is attached to the C ring, catechins in tea plants have been divided into non-esterified catechins including catechin (C), epicatechin (EC), epigallocatechin (EGC) and esterified catechins including catechin gallate (CG), gallocatechin gallate (GCG), epicatechin gallate (ECG), epigallocatechin gallate (EGCG) [6]. As the major monomeric catechin in tea [7], EGCG is important for increasing the health benefits quality and flavor quality of tea [8, 9], such as cancer prevention [10–12], and blood sugar control [13].

Light is the core environmental factor affecting the growth and quality of tea [14, 15], As the necessary factor for all plant function, light affected flavonoid biosynthesis, which can protect plants under the high light conditions [16]. Significant advances have been made in the research on the enhancement of flavonoids, phenols and anthocyanins by high light intensity [17–24], whereas decreasing of those by shading treatments [25–27]. Previous study showed that the catechins content of tea plants at low altitudes, in summer and at the plains is higher than that at high altitudes, in other seasons and at the hills [28–32]. Light was important for catechins biosynthesis [33], and the catechins content decreased under darkness and shading treatments [14, 34–40], indicating that the lower light intensity probably limits the catechins accumulation.

The genes involved in catechins biosynthesis are related to the phenylalanine pathway, flavonoid pathway and shikimic acid pathway [41, 42]. Light intensity regulates gene expression to affect the catechins content. For example, shading repressed the expression of genes in the flavonoid pathway such as *F3H, F3’H* and *DFR* in tea plants [43], while light induced the expression of *CHS, DFR1, DER2, ANS* and *ANR1* [44]. Meanwhile, the expression pattern of catechines-related genes (*ANR, ANS, LAR, C4H, PAL, CHI, CHS* and *DFR*) were related to high light intensity in summer [39]. However, the responses of catechins-related genes to light intensity changes have been inconsistent in previous studies. *ANS* plays a critical role in catechin biosynthesis in tea plants [45]: low light induces *ANS1* expression in safflower flowers, whereas high light upregulated *ANS* expression in red leaf lettuce [46, 47]. The response of *CsANS* expression to light is also inconsisitent [35, 43]. We speculate that genes also are regulated by other environmental factors that are dynamic in the field.

Catechins content is also affected by other environmental factors in the field, and there remains relatively few studies regarding the response mechanisms of catechins biosynthesis in tea plants to light intensity. Our research used the same of environmental conditions between for five light treatments (150, 250, 350, 450, 550 μmol·m^− 2^·s^− 1^) to explore: 1) the effect of light intensity to catechins content; 2) the response of catechins biosynthesis to light intensity; and 3) the relationship between catechins content and photosynthesis.

## Methods

### Plant materials

Tea seedlings (*Camellia sinensis* (L.) O. Kuntze), which were one-year-old cuttings of the Huangdan cultivar, were purchased from the Qianhe Tea Cooperative, Anxi County, China. The seedlings were transplanted into breeding bags (diameter = 16 cm and height = 18 cm), and cultivated in the nursery of Sino-Science Photobiotech Co. from March to August 2019. The composition of the culture medium was coconut bran, peat soil, vermiculite and perlite, and the volume ratio was 2:2:1:1. The composition of the culture medium was optimised by the Sino-Science Photobiotech Co. to provide a good growth environment for the roots of tea plants. In September, we transported the seedlings indoors to adapt to the environment of the plant growth facility, and the culture conditions are described in the Supplementary Table 1. Three biological replicates, including 120 plants that were clipped according to a standard procedure, were randomly distributed among the light treatments on October 1, 2019. Half of the samples were dried in an oven in two stages (120 °C10 min, 90 °C30 min) and stored at − 20 °C, and the other half were immediately frozen in liquid nitrogen and stored at − 80 °C until further experiments.

### Cultivation conditions

The tea seedlings used in this study were cultivated in a series of phytotrons under different conditions as shown in Supplementary Table 1. The nutrient solution was optimised as provided in Shigeki Konishi, Miyamoto, and Taki (1985), [48], and was provided by Sino-Science Photobiotech Company.

### Photosynthetic pigments and parameters

The pigment content was measured by 95% ethanol extraction. Fresh leaves (0.2 g, accurate to 0.0001 g) were ground in a centrifuge tube, and then 25 ml 95% ethanol was added. The mixtures were placed in the dark for 24 h and shaken every 5-6 h. The supernatant was extracted and analysed with a spectrophotometer (Mapada, China), and the absorbance at the following wavelengths was recorded: 665 nm, 649 nm and 470 nm.

Chlorophyll fluorescence was measured with an Imaging-PAM (Heinz Walz, Effeltrich, Germany). Tea plants were dark-adapted for 30 min before the measurement, and the third mature leaf from the top was used for the measurements. The minimum fluorescence (Fo) and maximum fluorescence (Fm) were obtained by applying measuring light pulses at a low frequency. The yield of variable fluorescence (Fv) was calculated as Fm − Fo, from which the maximum PSII quantum yield (Fv/Fm) was automatically calculated by the ImagingWin software (Walz). Other parameters (NPQ, qP, ETR, Fm′, Fo’) were also automatically calculated by the ImagingWin software (Walz).

The photosynthesis characteristics including net photosynthetic rate (Pn), transpiration rate (Tr), stomatal conductance (Gs), and intercellular CO2 concentration (Ci), were measured using a Li-6800 portable photosynthesis system (LI-COR Inc., Lincoln, NE, USA). All measurements were conducted from 10:00 to 12:00 am. The irradiance and temperature of the leaf chamber were set according to the conditions in each treatment, and the results were recorded when Pn reached a steady state.

### Catechin content determination

The catechins content determination was performed according to the method described by Lin et al. (2017) [49]. Catechins were determined by HPLC. Tea samples (0.2 g, accurate to 0.0001 g) were weighed into a 10 mL centrifuge tube, and 5 ml of 70% methanol solution heated in a water bath was added. After being shaken by a mixer, the tea was immediately transferred to a 70 °C water bath. The tea was immersed for 10 min and shaken once at 5 min, and the centrifuge tube was transferred to the centrifuge after 10 min (3500 r/min, 10 min). The residue combined with 5 ml of 70% methanol solution was extracted once, and the procedure was repeated as above. The combined extract volume was brought to 10 mL, shaken, and pour 1 ml liquor into a 10 mL volumetric flask with a pipette and brought to 10 mL with a stable solution. The mixture was then filtered with 0.45 μM membrane before being analysed by HPLC. The HPLC instrument was a Waters Acquity UPLC HSS T3 column (2.1 *100 mm, RP181.7 um) with a 35 °C column temperature.

Mobile phase A:100% pure water + 0.02% EDTA-2Na + 2% glacial acetic acid; Mobile phase B: 100% acetonitrile + 2% glacial acetic acid. PDA detection conditions: scanning range of 200 nm-400 nm, characteristic detection wavelength of 278 nm, scanning time of 10 min, injection volume of 2 ul. Standard stock solution is listed as follows: caffeine stock solution 2.00 mg/ml, gallic acid (GA) stock solution 0.100 mg/ml, catechin stock solution: C 1.00 mg/ml, EC 1.00 mg/ml, EGC 2.00 mg/ml, EGCG 2.00 mg/ml, ECG 2.00 mg/ml. All samples were analysed with three biological replicates, and the average values were used for data analysis.

### RNA extraction and quantitative RT-PCR

Total RNA was isolated using the RNAprep Pure Plant Kit (DP441, TIANGEN Company, China) according to the operating instructions. First-strand cDNAs were synthesized using FastKing gDNA Dispelling RT SuperMix (KR118, TIANGEN Company, China). The qRT-PCR analyses were carried out on an ABI 7500 HT Real-time PCR system (ABI Company, USA). The PCR reaction conditions were as follows: incubation at 95 °C for 15 min, 40 cycles at 95 °C for 10s, and annealing at 61 °C for 32 s, in triplicate for each reaction. All primers used for qRT-PCR are listed in Supplementary Table 2, and ***β-****Actin* was used as an endogenous control in this assay. The expression levels of related genes were calculated using the 2^−ΔΔCt^ method.

### Statistical analyses

One-way analysis of variance was used to determine significance, and Duncan’s multiple range test was used to evaluate differences between groups with SPSS 21.0 statistical software. This software was also be used for the correlation analysis and regression analysis between the content of catechins and other variables. The data are presented as the means ± standard deviations from three biological replicates, and the different letters represent significant differences between groups (lowercase letters represent a significant difference, and uppercase letters represent an extremely significant difference).

Principal component analysis (PCA) of nine morphological traits was performed using the SIMCA 13.0 software with the nine variables as the primary ID and the treatments as the second ID. The figure legend indicates the representation of the three biological replicates.

Partial least square (PLS) analysis was performed using the Minitab 16 software, and the confidence level was set to 95%. The r-squared value represents the degree of fitting between the measured ranking and predicted ranking in PLS.

## Results

### Light intensity controlled catechins accumulation and related gene expression

When tea plants were grown in the same environment at different light intensities, the contents of EGCG, gallic acid (GA) and total catechins (TC) under an appropriately high light intensity (250 μmol·m^− 2^·s^− 1^) were significantly higher than those under extremely high and low light intensities (550 μmol·m^− 2^·s^− 1^ and 150 μmol·m^− 2^·s^− 1^), which suggested that an appropriately high light intensity promoted catechins accumulation.

**Fig. 1:**
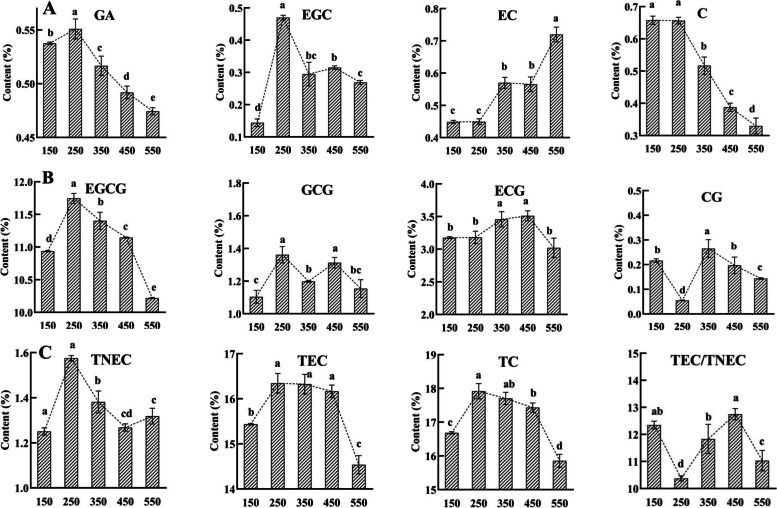
Catechins content is controlled by light intensity. **A**, **B** and **C** represent the content non-esterified catechins, esterified catechins and total catechins, respectively.150,250,350,450,550 represent the treatment of 150, 250, 350, 450, 550 μmol·m^− 2^·s^− 1^ light intensity

The contents of the precursor of esterified catechins biosynthesis, non-esterified catechins and gallic acid (GA), were the highest in the 250 μmol·m^− 2^·s^− 1^ treatment, and the contents of gallic acid (GA), total non-esterified catechins (TNEC), EGC and C were 16.16, 74.73, 98.94 and 19.50% higher than those in 550 μmol·m^− 2^·s^− 1^ treatment, respectively (Fig. 1). When grown under the different light intensity treatments, the contents of EGCG, total esterified catechins (TEC) and total catechins (TC) in tea plants exhibited a similar response pattern, reaching maxima in the highest in 250 μmol·m^− 2^·s^− 1^ treatment, decreased with the 350, 450 and 150 μmol·m^− 2^·s^− 1^ treatments, and reaching minima in the lowest in 550 μmol·m^− 2^·s^− 1^ treatment. These results implied that appropriately high light increased the catechins content by enhancing the contents of the precursor of esterified catechins biosynthesis.

Similar to the accumulation patterns of catechins content, the expression of 14 catechins-related genes (*CsPAL, CsC4H, Cs4CL1, Cs4CL2, CsF3’H, CsLAR1, CsLAR2, CsDFR1, CsANR2, CsSCPL, CsaroB, CsaroDE1, CsaroDE2* and *CsaroDE3*) was the highest in the 250 μmol·m^− 2^·s^− 1^ treatment but was suppressed in the extremely high and low light intensity (550 μmol·m^− 2^·s^− 1^ and 150 μmol·m^− 2^·s^− 1^). Notably, the response patterns of 8 catechins-related genes to the light intensity changes were similar to those of the content of EGCG, TEC and TC (Fig. 6, Supplemental Fig. 2). These results demonstrated that appropriately high light intensity increased the catechins content by inducing the expression of catechins-related genes, whereas extremely high or low light intensity suppressed those expressions and decreased catechins content.

### Light intensity controlled photosynthetic capacity and related gene expression

**Fig. 2:**
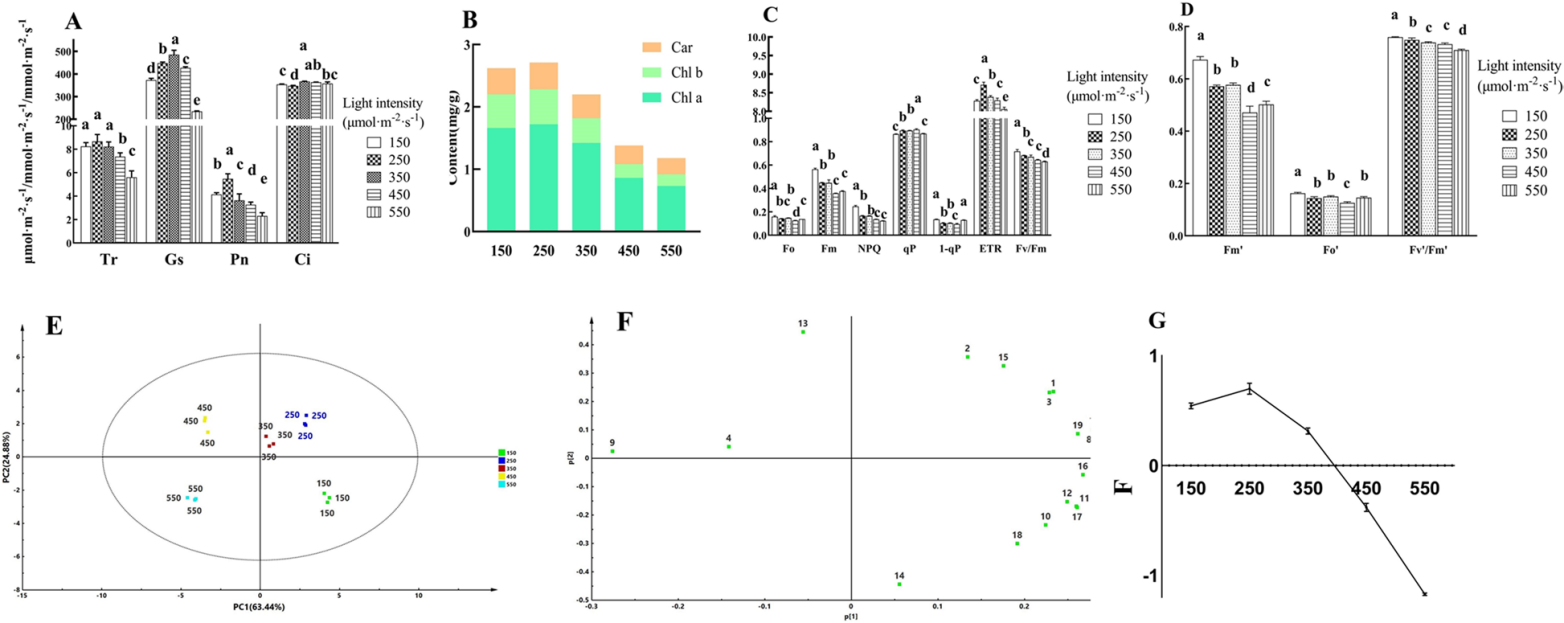
Photosynthesis capacity is controlled by light intensity. **A** and **B** represent the photosynthesis parameters and photosynthetic pigment content; **C** and **D** represent chlorophyll fluorescence under different light intensities. E is the score plot of PCA, and 150,250,350,450,550 represents the treatment of 150, 250,350,450,550 μmol·m^− 2^·s^− 1^ light intensity. F is the loading plot of PCA,and the corresponding indexes of the numbers are as follows: 1 = Pn, 2 = Tr, 3 = Gs, 4 = Ci, 5 = Chla, 6 = Chlb, 7 = Car, 8 = Chla+b, 9 = Chla/b, 10 = Fo, 11 = Fm, 12 = NPQ, 13 = qP, 14 = 1-qP, 15 = ETR, 16 = Fv/Fm, 17 = Fm′, 18 = Fo’, 19 = Fv’/Fm′. G represents photosynthesis capacity

**Fig. 3:**
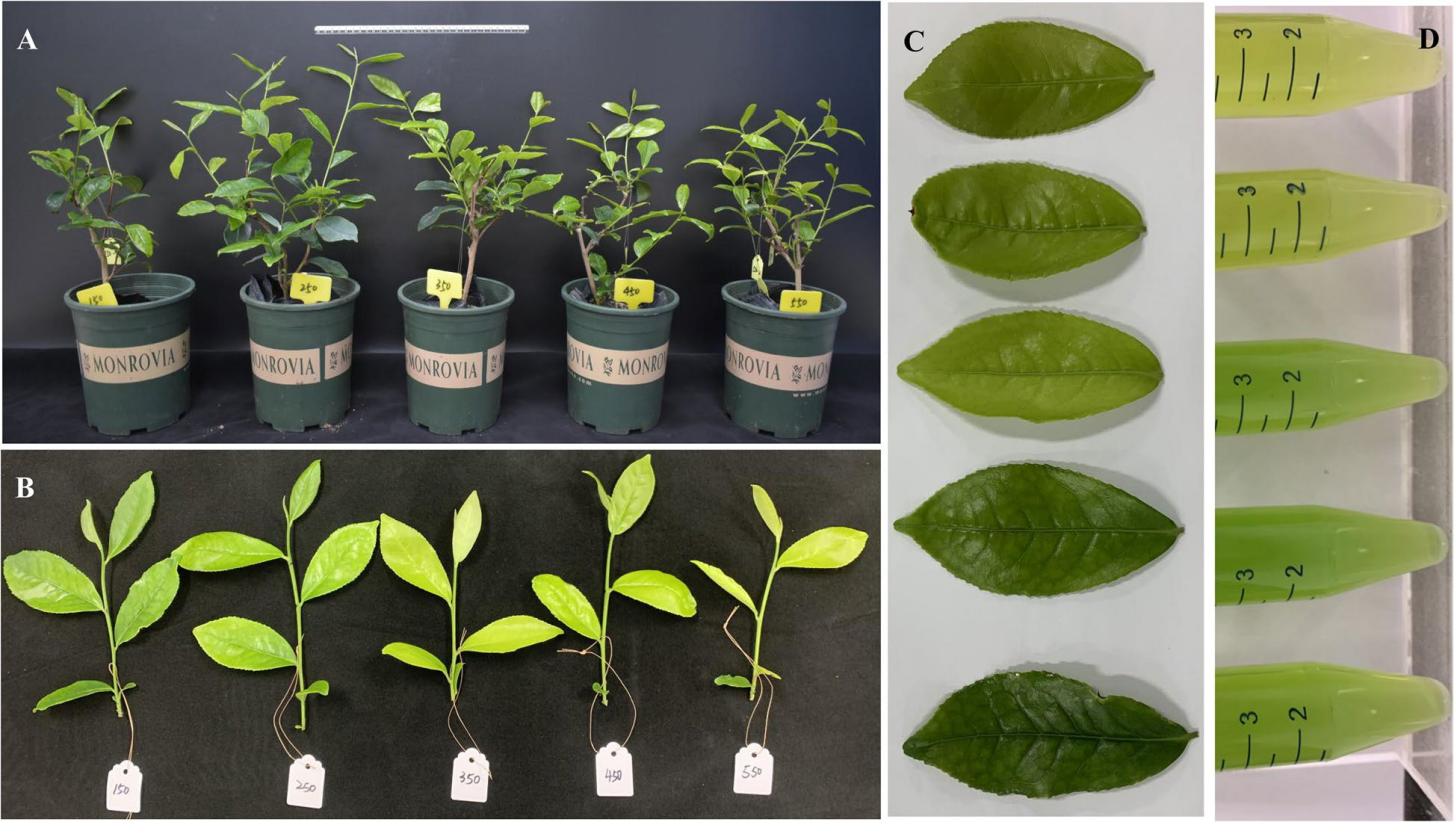
Phenotypes of the tea plant under different light treatments. From left to right and from top to bottom, they represent the treatment of 150, 250,350,450,550 μmol·m^− 2^·s^− 1^

Photosynthesis-related parameters were significantly affected by light intensity. Appropriately high light (250 μmol·m^− 2^·s^− 1^) resulted in the highest photosynthetic rate (Pn), transpiration rate (Tr) and electron transport rate (ETR), which were 130.49, 53.88 and 8.10% higher than those at 550 μmol·m^− 2^·s^− 1^, respectively (Fig. 2A, C). The chlorophyll a (Chla), chlorophyll b (Chlb) and carotenoid (Car) contents increased under the 150 μmol·m^− 2^·s^− 1^ and 250 μmol·m^− 2^·s^− 1^ treatments but decreased under the 450 μmol·m^− 2^·s^− 1^ and 550 μmol·m^− 2^·s^− 1^ treatments, which was in agreement with the observed leaf colour of the tea plants (Fig. 2B, Fig. 3). All of the photosynthesis-related parameters decreased in the 550 μmol·m^− 2^·s^− 1^ treatment, but chlorophyll fluorescence parameters were higher in the 150 μmol·m^− 2^·s^− 1^ treatment. These results suggested the accelerated photosynthesis of tea plants under appropriately high light.

The 19 photosynthesis-related parameters were analysed by PCA, and the cumulative contribution rate of the first three principal components were 95.49% (F1, F2, F3 and F represent the score of principal components 1, 2, 3 and the comprehensive evaluation in the model). The scattered tea samples grown under the different light intensities in the PCA score plot and the score of comprehensive evaluation (F) in the model demonstrated the diverse photosynthesis capacities of the different treatments (Fig. 2E). F was the highest in the 250 treatment but gradually decreased with the increasing light intensity from the 350 μmol·m^− 2^·s^− 1^ to 550 μmol·m^− 2^·s^− 1^ treatment (Fig. 2G), which meant extremely high light intensity exceeding the light saturation point inhibited photosynthesis of tea plants. Accordingly, we deduced that appropriately high light enhanced the photosynthetic capacity of tea plants.

Similar to the response pattern of the photosynthetic capacity, appropriately high light (250 μmol·m^− 2^·s^− 1^) upregulated the expression of 9 photosynthesis-related genes (*CspsbA, CspsbB, CspsbC, CspsbD, CsPsbR, CsGLK1, Cschl, CsCHLG, bchG, CsRCA* and *CsPETD;* Fig. 6A, Supplemental Fig. 2), and the response patterns of 6 genes expression were similar to those of Pn and Tr. Thus, appropriately high light resulted in higher photosynthetic capacity by upregulating the expression of related genes, whereas the suppressed expression of those genes resulted in slower photosynthesis in tea plants under excessively high light intensity.

### Photosynthetic capacity positively regulated the response of catechins biosynthesis to light intensity in tea plants

#### Catechins accumulation was related to photosynthetic capacity

The results of regression and correlation analysis indicated that the catechins accumulation and photosynthetic capacity of tea plants under different light intensities were closely related: the contents of EGCG, TEC and TC were regulated by photosynthetic capacity, while the non-esterified catechins content was significantly related to the photosynthetic pigments content.

**Fig. 4:**
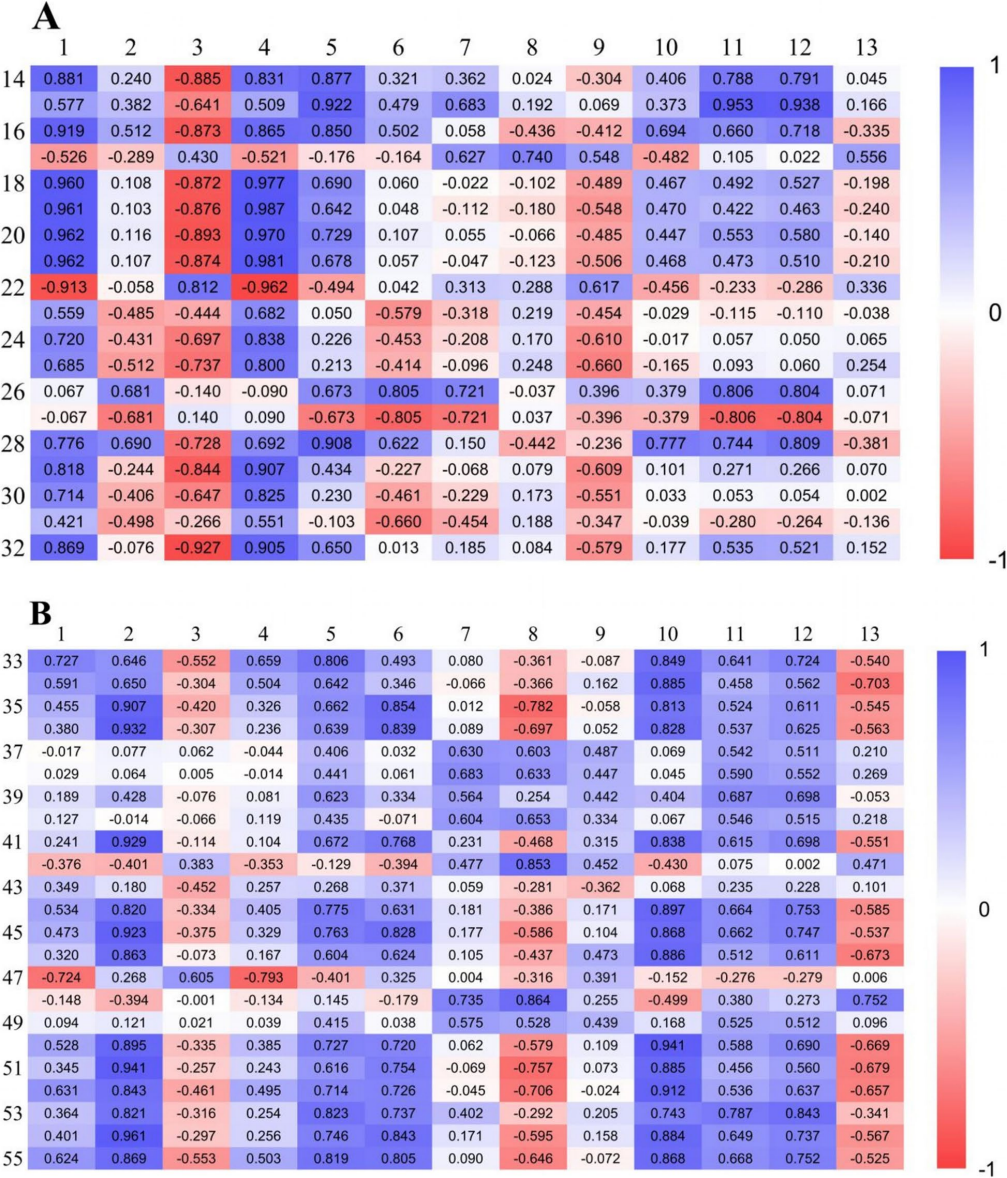
The correlation between catechins content and photosynthesis-related indexes and catechins-related genes expression, and the corresponding indexes of the numbers were shown in the supplemental data 2

**Fig. 5:**
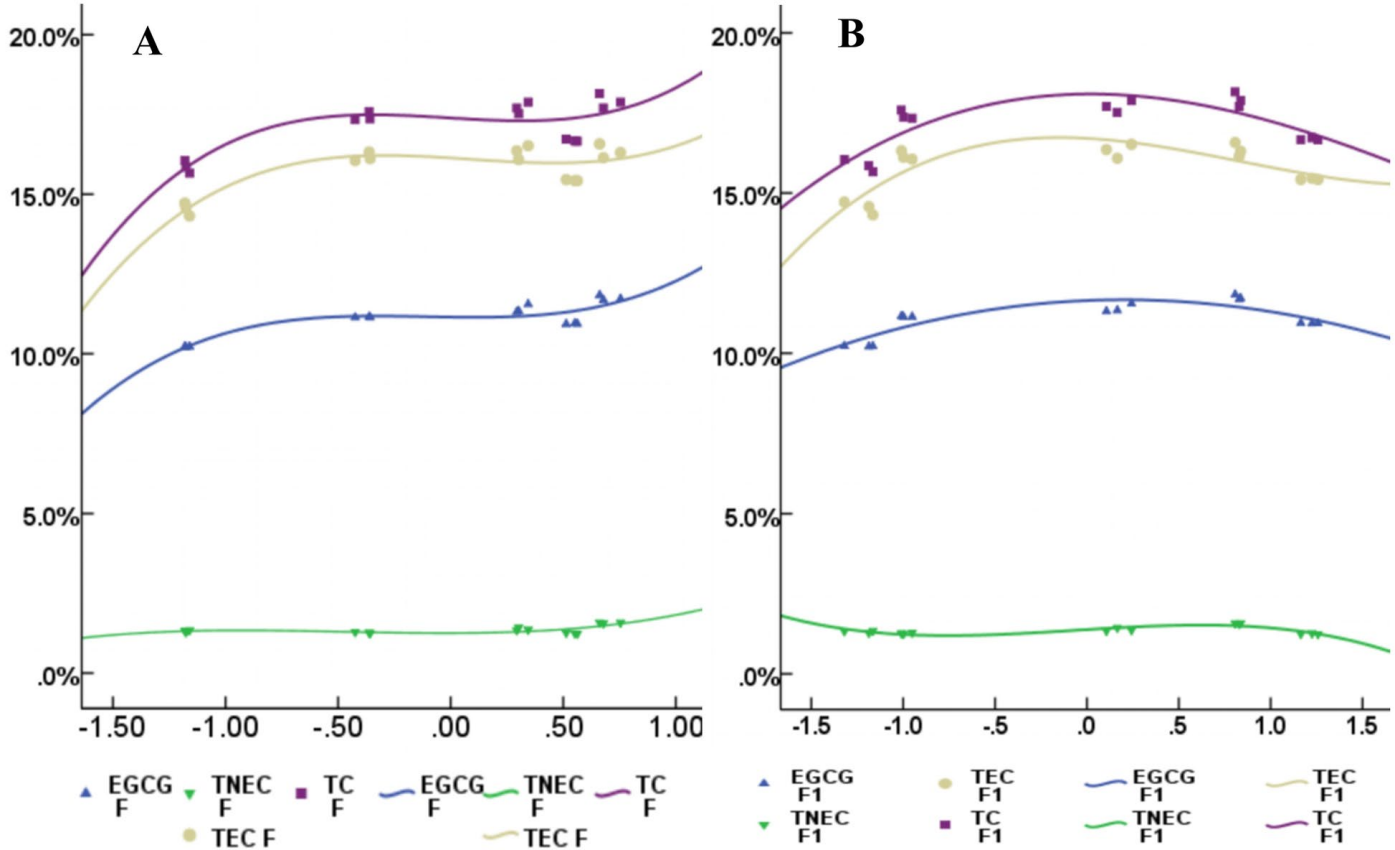
The curve fitting between catechins content and photosynthesis capacity under different light intensities. **A** represents the curve fitting between F and EGCG, TNEC, TC respectively; **B** represents the curve fitting between F1 and EGCG, TNEC, TC respectively

Correlation analysis suggested that the precursors for synthesizing esterified catechins (GA and C) were significantly and positively related to the photosynthetic pigment content (Chla, Chlb, Car and F1), but the contents of EGCG, TEC and TC were significantly and positively related to chlorophyll fluorescence (qP, ETR, Fv’/FM’) and photosynthetic capacity (Fig. 4A). Meanwhile, a cubic function described the relationship between the contents of EGCG, TEC, TC and F, F1, but the R^2^ of the equation between TNEC and F1 (67.0%, Fig. 5B) was higher than that between TNEC and F (47.9%, Fig. 5A, Supplemental Table 3), indicating that esterified catechins were affected by photosynthetic capacity, whereas non-esterified catechins were mostly regulated by photosynthetic pigments. Using partial least squares analysis, a high degree of model fit could be detected between catechins content and photosynthetic parameters (R^2^ > 80%, Supplemental Fig. 1), which suggested that the response of catechins content, especially EGCG to light intensity was related to the photosynthetic capacity of tea plants.

### Photosynthesis-related genes and catechins biosynthesis-related genes co-regulated the response of catechins accumulation to light intensity

The response of catechins-related genes to light intensity was regulated by gene expression related to photosynthetic capacity. The expression of 34 photosynthesis- and catechin-related genes was the highest under the 250 or 350 treatment, and the expression patterns under different treatments of 4 photosynthesis-related genes (*CspsbE, CspsbC, CspsbD, CsCHLG, bchG*) were similar to those of 3 catechins-related genes (*CsCHI, CsaroB, CsaroDE3*).

**Fig. 6:**
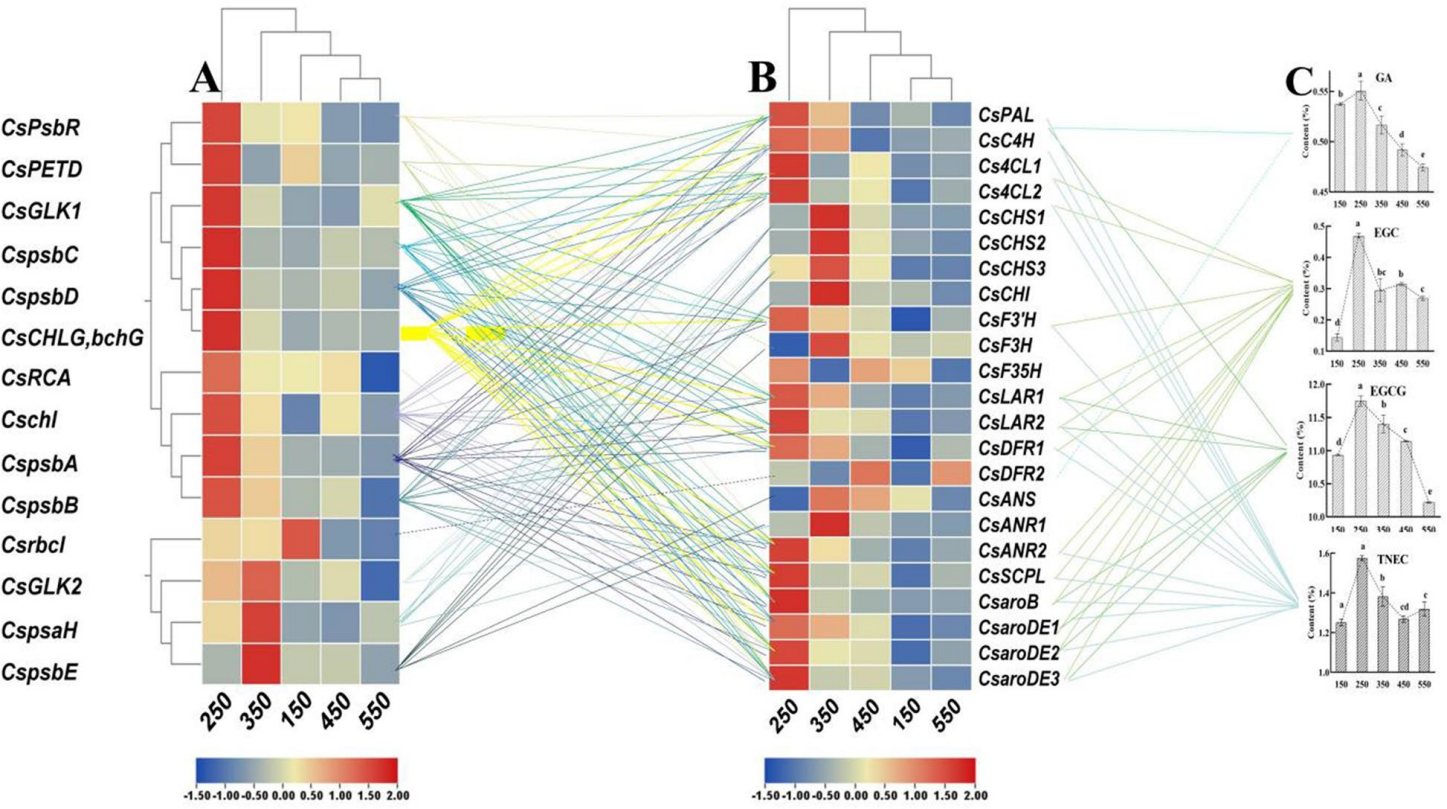
Gene related to photosynthesis capacity regulated the expression of catechins-related genes and then mediated the response of catechins content to light intensity. **A**, **B**, and **C** represent genes expression related to photosynthesis capacity, catechins-related genes expression, and the catechins content. Solid lines represents the person> 0.7 and significance< 0.005, and the dotted line represents the person<− 0.7 and significance< 0.005

The expression levels of catechins-related genes involved in the phenylalanine pathway (*CsPAL, CsC4H, Cs4CL1, Cs4CL2*) were positively related to PSII-related genes (*CspsbA, CspsbB, CspsbC, CspsbD, CspsbR*) and ETR-related genes (*CsPETD*), clustering in one group (Fig. 6A, B, supplemental Fig. 2). Moreover, the expression levels of catechins-related genes in the downstream of flavonoid pathway (*CsLAR2, CsDFR1, CsDFR2, CsANS, CsANR2*) and in the shikimic acid pathway (*CsaroB, CsaroDE1, CsaroDE2, CsaroDE3*) were positively correlated with those of chlorophyll-related genes (*CsGLK1, CsGLK2, CsCHLG, bchG*). In contrast, the expression levels of *CsRCA* and *Csrbcl* were negatively related to *CsF35H* and *CsANR1*, respectively.

Gene expression was related to catechins biosynthesis, which was regulated by photosynthesis-related genes and thereby controls catechins accumulation (Fig. 6C). For example, the contents of EGCG, TEC and TC were positively regulated by the expression of catechins-related genes in the phenylalanine pathway (*CsPAL, Cs4CL2, CsCHS2, CsCHS3, CsCHI*), the downstream of flavonoid pathway (*CsLAR2, CsDFR1, CsDFR2, CsANS, CsANR2*) and the shikimic acid pathway (*CsaroB, CsaroDE1, CsaroDE2, CsaroDE3*).

### Mechanisms underlying photosynthesis-regulated catechins accumulation

**Fig. 7:**
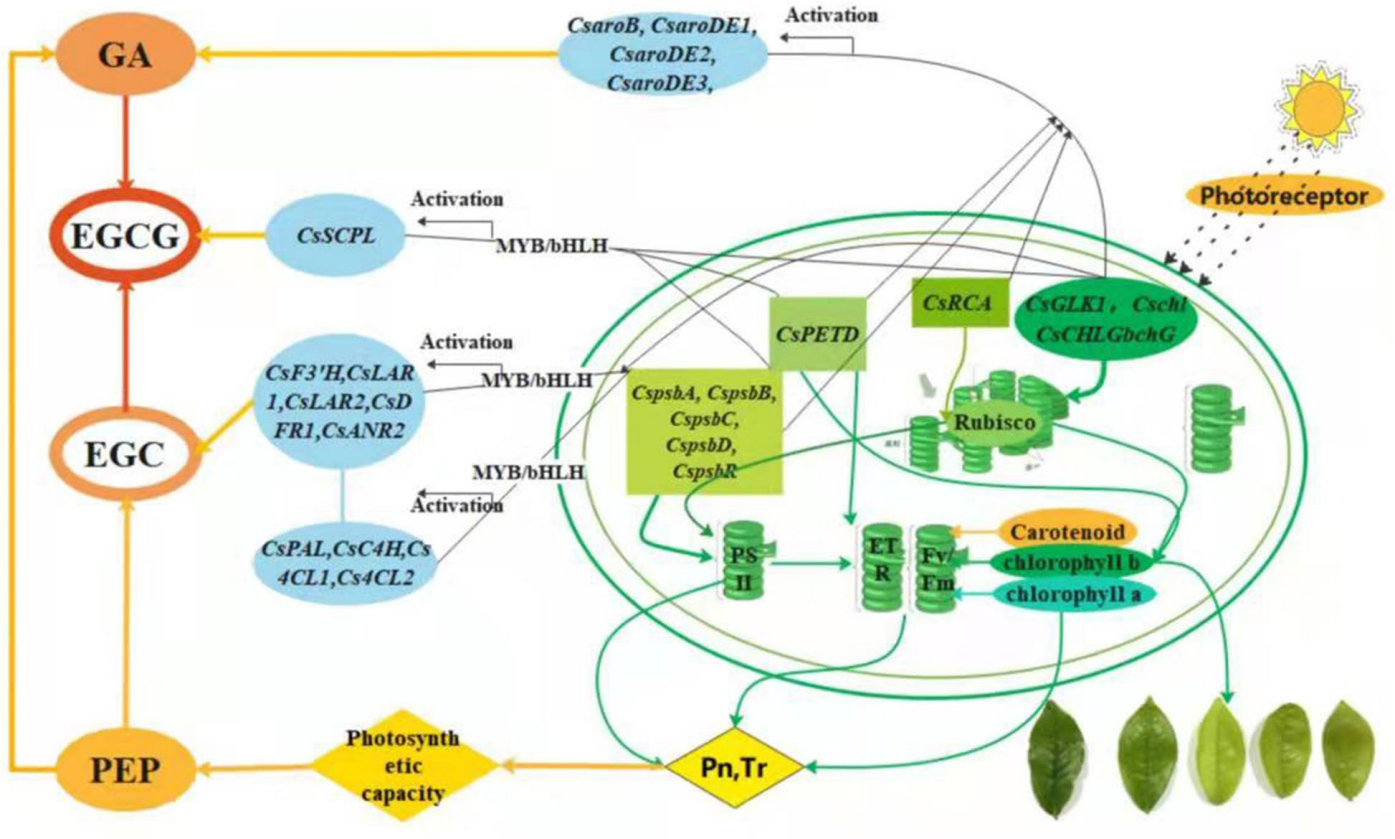
The mechanism of the response of catechins biosynthesis to light intensity changes by photosynthesis capacity. Pn = Net photosynthetic rate; Tr = Transpiration rate; EGC = Epigallocatechin; GA = Gallic acid; EGCG = Epigallocatechin gallate; PEP = phosphoenolpyruvate

Photosynthate is the substrate for catechins synthesis. On the one hand, the expression of photosynthesis-related genes affected the photosynthesis capacity of tea plants, including Pn, Tr, and ETR, and thereby regulated the content of substrate for catechins biosynthesis. On the other hand, genes related to photosynthetic capacity regulated the expression of catechins-related genes by the promoter of structural genes and transcription factors and thereby affected the catechins content of tea plants (Fig. 7).

The appropriately high light intensity upregulated the expression of genes related to PSII (*CspsbA, CspsbB, CspsbC, CspsbD, CsPsbR*), photosynthetic pigments (*CsGLK1, Cschl, CsCHLGbchG*), electron transfer rate (*CsPETD*), and Rubisco activity (*CsRCA*), which enhanced the photosynthetic capacity of tea plants, including the net photosynthetic rate (Pn), transpiration rate (Tr), and electron transfer rate (ETR). On the other hand, the genes related to PSII and photosynthetic pigments regulated the the expression of genes related to non-esterified catechins biosynthesis (*CsPAL, CsC4H, Cs4CL1, Cs4CL2*, *CsF3’H, CsLAR1, CsLAR2, CsDFR1, CsANR2*). These results suggested that the substrate content (such as PEP) and the expression of genes related non-esterified catechins biosynthesis, which were regulated by the genes related to photosynthesis capacity, co-affected the content of non-esterified catechins (such as EGC). Meanwhile, these photosynthesis-related genes also regulated the expression of gallic acid (GA) biosynthesis genes in the shikimic acid pathway (*CsaroB, CsaroDE1, CsaroDE2, CsaroDE3*) to affect the GA content. Eventually, the increased contents of the precursor of esterified catechins (EGC and GA) and the upregulation of *CsSCPL* were mediated by the photosynthetic capacity and the related genes in the tea plant, which promoted the accumulation of esterified catechins (such as EGCG).

In contrast, the low photosynthetic capacity induced by downregulated expression of photosynthesis-related genes, which suppressed the expression of catechins-related genes, and accordingly decreased catechins accumulation in tea plants under extremely high and low light intensity.

## Discussion

### Light controlled catechins accumulation in tea plant

In this study, the catechins content was enhanced by appropriately high light intensity but decreased under extremely low or high light intensity. The pathways of flavonoid synthesis, catechins polymerization and flavonols glycosylation were regulated by light [35, 50, 51], so we speculated that the decreased catechins content under low light intensity was due to the increased content of flavone or anthocyanin; this was consistent with the more purple bud colour in the 150 treatment. Catechin monomers responded differently to different light intensities in our research, and a previous study found that epicatechins declined but catechins was elevated in the dark [50], probably because the hydroxylation of B ring is related to the light signal [34].

**Fig. 8:**
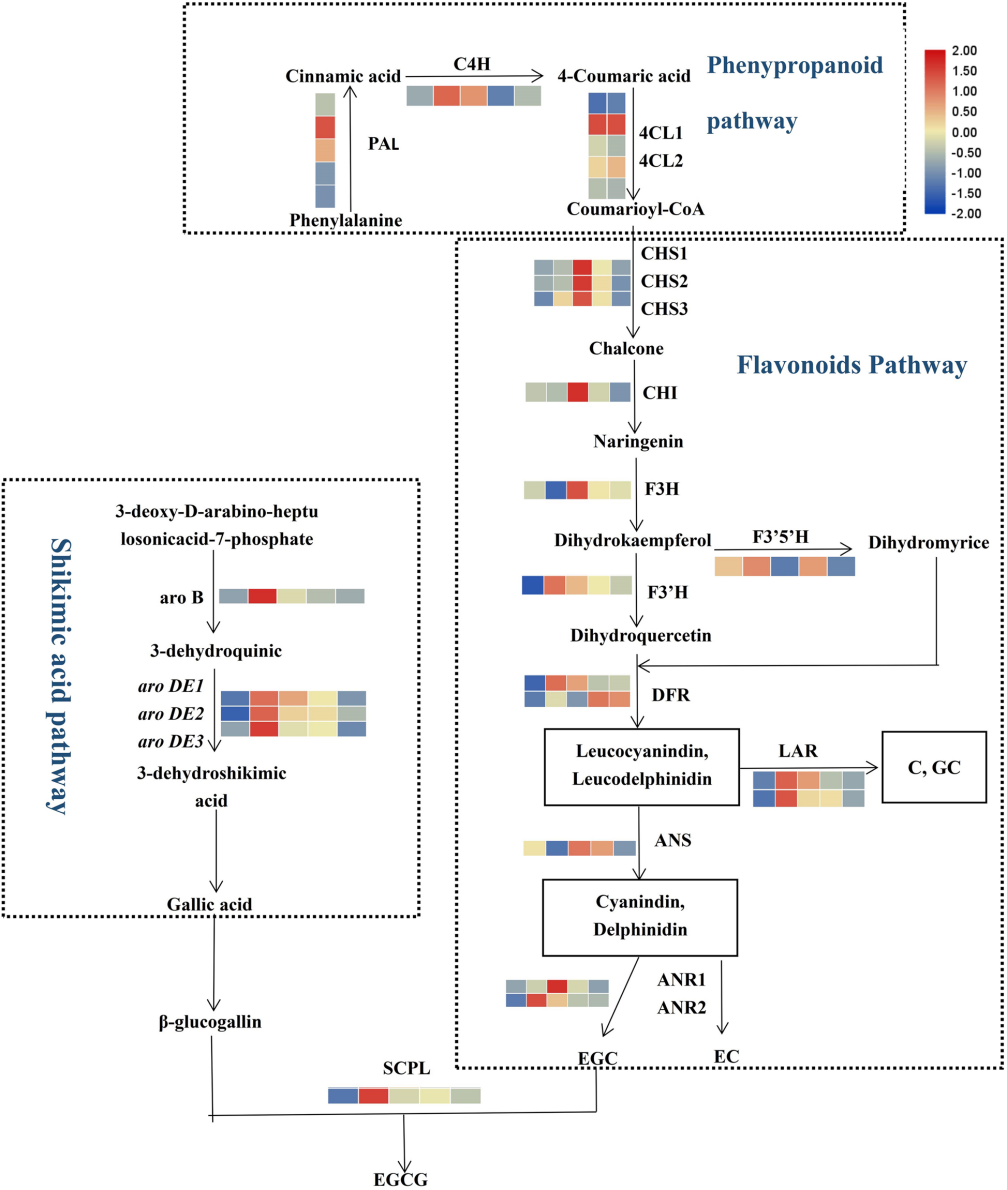
The regulation pathway of catechins-related genes under different light intensities

Catechins biosynthesis is involved in the shikimic acid pathway, phenylalanine pathway and flavonoid pathway. Gallic acid and non-esterified catechins are the precursors of esterified catechins [52]. The expression levels of *CsF3’ H* and *CsDFR* in the flavonoid pathway decreased rapidly in the shading treatment, resulting in decreased catechins content [43], which was consistent with the decreased catechins obtained by suppressing the expression of catechins-related genes under low light in our study (Fig. 8). The expression patterns and functions of *CsDFR1* and *CsDFR2* varied in our study, probably because the *CsDFRs* gene family is differently related to specific metabolites [32].

### Catechins accumulation of tea plant were related to photosynthesis capacity

The flavonoids in tea plants, including catechins, flavonol and anthocyanin, and their biosynthesis require photosynthate as the substrate. Phosphoenolpyruvate (PEP) is essential for catechins biosynthesis. In the previous research, the flavonoids pathway was closely related to photosynthesis and chlorophyll content [53, 54], and there was the same response pattern between the content of total flavone and Pn when the light intensity changed [55]. Catechins biosynthesis in tea plants seriously was affected by photosynthesis [56], as well as the daily course of photosynthetic activity of tea leaves and the accumulation of catechins in tea leaves have almost parallel characteristics [57, 58]. Additionally, photosynthesis and chlorophyll influenced catechins biosynthesis and epicatechin content [59], which is consistent with our result that the catechins content was positively correlated with Pn and Tr.

We also found that the catechins content was related to Fm, ETR, FM’ and v’/FM’, and gene expression was related to photosynthetic capacity. A previous study has indicated that the differentially expressed genes between the normal plant and pale green mutant were mainly involved in photosynthesis and flavonoids pathway. Other environmental factors regulating the secondary metabolism of tea plants were dynamic under field conditions, and we confirmed the precise responses of catechins to light intensity and its underlying mechanism using phytotron. In summary, our results indicated that photosynthetic capacity controls catechins biosynthesis by regulating the substrate content and the expression of catechins-related genes when tea plants are grown under different light intensities.

### The inhibition of catechin biosynthesis under the extreme light intensity was related to shoot growth of tea plant

Interestingly, consistent with the responses of catechins and photosynthetic capacity to light intensity changes, the young shoots grew slowly under excessively low or high light but grew more quickly under appropriately high light. We speculated that the regulation of catechins by light intensity was affected by shoot growth. We previously observed that catechins biosynthesis responded to temperature changes through shoot growth [60]. The higher chlorophyll and theanine contents but lower flavonoid contents under a shading treatment may have been related to the lower growth rate of young shoots [61]. High light intensity reduced growth and altered the catechins content of tea plants [62]. Therefore, the combined effects of the slow shoot growth and the decreased photosynthetic capacity inhibited catechins accumulation under excessive light intensity. We will test this hypothesis in future research. Future studies should also examine how other aspects of light conditions such as light quality and photoperiod influence catechins biosynthesis.

## Conclusions

The catechins content and photosynthetic capacity of tea plants increased under appropriately high light intensity but decreased under the the extremely low or high light intensity. Photosynthesis- and catechins-related genes exhibited the same response pattern under the different light intensities. Based on PCA, correlation analysis and regression analysis, we found that catechins accumulation was affected by photosynthesis, and catechins-related genes were regulated by photosynthesis-related genes. In summary, the control of catechin accumulation by light in tea plants is mediated by the plant photosynthetic capacity. The research provided useful information for improving catechins content and its light-intensity regulation mechanism in tea plant.

## Supplementary Information

**Additional file 1: Supplemental Table 1**. Culture conditions of tea plants.

**Additional file 2: Supplemental Table 2**. Primer sequences used for reverse transcription-quantitative PCR.

**Additional file 3: Supplementary Table 3**. The cubic function equations in curve-fitting analysis.

**Additional file 4: Supplemental Figure 1**. The PLS analysis between catechins content and the indexes of photosynthesis capacity of tea plants under different light intensity.

**Additional file 5: Supplemental Figure 2**. The expression of related genes under the different light intensity and their clustering.

**Additional file 6: Supplemental data 1**. The corresponding indexes of the numbers in the correlation analysis.

## Data Availability

The datasets used and/or analysed during the current study available from the corresponding author on reasonable request (ljk213@163.com).

## Abbreviations

EGCG: Epigallocatechin gallate
CG: Catechingallate
GCG: Gallocatechin gallate
ECG: Epicatechingallate
C: Catechin
EC: Epicatechin
EGC: Epigallocatechin
GA: Gallic acid
Pn: Net photosynthetic rate
Tr: Transpiration rate
ETR: Electron transfer rate
Gs: Stomatal conductance
PCA: Principal component analysis
PLS: Partial Least Square
TC: Total catechins
TNEC: Total non-esterified catechins
TEC: Total esterified catechins
