# Supplementary material for: Light control of catechin accumulation is mediated by photosynthetic capacity in tea plant (Camellia sinensis)
Source: BMC Plant Biol. 2021 Oct 20;21:478. doi: 10.1186/s12870-021-03260-7 (PMC8527772; doi:10.1186/s12870-021-03260-7)
Supplement: Supplementary file 1 — Additional file 1: Supplemental Table 1. Culture conditions of tea plants. [file 12870_2021_3260_MOESM1_ESM.doc]

Supplementary table 1. Culture conditions of tea plants

| Environmental factors | Adaption period  (September 2019) | Treatment period  (October 1 to December 31 in 2019) | |
| --- | --- | --- | --- |
| Light intensity | 200µmol·m^-2^·s^-1^ | | 150, 250, 350, 450,550µmol·m^-2^·s^-1^ |
| Temperature | 25±2℃ | | 30±2℃ |
| Air humidity | 70±5% | | 90±5% |
| Medium relative humidity | 70%-85% | | 80% |
| Nutrient solution | Watering 100ml solution every five days，EC=1.2 | | Watering 60ml solution every five days，EC=1.5 |
| The relative water content of medium | 80±2% | | |
| Photoperiod | 12h:12h | | |
| Light quality | Model number: ZK-SL200-0S01/; The ratio of blue and red = 3.7:1 | | |
| CO^2^ concentration | 750±50 µmol·mol^-1^ | | |
