# Supplementary material for: Light control of catechin accumulation is mediated by photosynthetic capacity in tea plant (Camellia sinensis)
Source: BMC Plant Biol. 2021 Oct 20;21:478. doi: 10.1186/s12870-021-03260-7 (PMC8527772; doi:10.1186/s12870-021-03260-7)
Supplement: Supplementary file 2 — Additional file 2: Supplemental Table 2. Primer sequences used for reverse transcription-quantitative PCR. [file 12870_2021_3260_MOESM2_ESM.doc]

Supplementary table 2. Primer sequences used for reverse transcription-quantitative PCR

| Gene name | Primer sequence (5’→3’) |
| --- | --- |
| *CsaroB* | F:CCTTGGTGGTGGTGTCATTG R:GTATCAGTATCTATAAGCACACATTC |
| *CsaroDE1* | F:CCTTCGACAAGCTTATGGAGTTG R:ACCTTTGCTGTGGCCCACT |
| *CsaroDE2* | F:CAACTTCGGATGTTCAAACAAGTAGT R:CCTTCGCCTTCCTCATATCTGT |
| *CsPAL* | F:GGGAGTCATTTGGAGGAAGT R:CCTCCTATGTGAAGTAGCACC |
| *CsC4H* | F:AACCACCGCAACTTGACC R:CCTCCAGTGCTCACCGT |
| *Cs4CL1* | F:GCACAATAACATCCCTCCG R:TCCAGACCTCAGCGAAGG |
| *Cs4CL2* | F:AGAGCGAAATCCCGACAGT R:TCCATCCACTTGTTGAGCC |
| *CsCHS1* | F:GTGATTGTGTTATGGGTTGGC R:GCAAAGAACATGTTATTTTCC |
| *CsCHS2* | F:GTTGCTCTATCATTGAACTAC R:GCAAACAACCAAAGCAAAG |
| *CsCHS3* | F:CAGCCACCACCCTAAACAT R:AACTCCACCTTATGCTCGC |
| *CsCHI* | F:TCTCTCTCCTAAACTCTCATC R:CATTTGTGGCTCTTCATCAG |
| *CsF3’5’H* | F:TCAACCAAGTAGTGCTTTGC R:TGAGTGATGAGGATGACGAG |
| *CsF3H* | F:CGGGAAGAAAGGTGGATTC R:TAGGTCTCCGTTACAGCCCT |
| *CsDFR1* | F:GGAAGGCGGATTTGAATG R:CACCAGCCTCTTCACTGTCT |
| *CsDFR2* | F:ATTGGCAGAGAAAGCAGCAT R:GTGATTAGGCTTGGTGGGAA |
| *CsLAR1* | F:CGTTGATAGGGCTAATCCG R:TGCTTTGACACTGCCATC |
| *CsLAR2* | F:AATTCACCATCAAAACCGTCGAC R:TCTTCTTCTCCCACAAAGATGCA |
| *CsANS* | F:GCTACCAAGACCCTCTCAAT R:AAGTCAGTGTGGGCTTCAA |
| *CsANR1* | F:TTGTGGCAGAGAAAGAATCG R:CCCATACTTGAAACTGAATCC |
| *CsANR2* | F:CAACAACAACCAAACCGATG R:CATCAGTTAGGTCTGCTCGG |
| ***β-****Actin* | F:CTTCCTCATGCTATCCTCCGTCTT R:ATTTCCCGTTCAGCAGTGGTG |
| *CsSCPL* | F:ATGGCAGAGAAGCAATATAACC R:CCAGAAGACACTACATGTGGAAAG |

Continue to Supplementary table 2

| Gene name | Primer sequence (5’→3’) |
| --- | --- |
| *CspsaH* | F: AGGACATCGGCAACACCAC R: CAGCAGGAACTTGAGCAGGA |
| *CspsbA* | F: CAGATTCGGTCAAGAGGAAGAA R: CCAAGCAGCCAAGAAGAAATG |
| *CspsbB* | F: CAGCTGCCCGGAGTCTATTT R: TACGAATCTCCTCAGCACGC |
| *CspsbC* | F: ACCTGCGTGCCTATGACTTC R: CGCCATCCAAGCACGAATAC |
| *CspsbD* | F: CGCAGTTTCTACTCCTGCTAAT R: AAGTCCACAGACCACCTAATTG |
| *CspsbE* | F: CCGGATTGGGTTCATCCCTG R: CCTTCCCCCTCGAATCAGAC |
| *CsPsbR* | F: ATTCAGGCCAGTGGTAAGAAGATC R: TCGAGTCCATCCGTCAAGTCC |
| *CsGLK1* | F: ATCATCCGCACAATCCAAGAATCCTCA R: GGTGGCAGCAGTGGTGGGTGTTTAG |
| *CsGLK2* | F: TTTGCTGGACAGTATTGATTTTGATGAC R: TTCTTAGATGATTTTCGGCCTTTATGAG |
| *Cschl* | F: CTTGGTTGTTGCTGTTGTTG R: GGTGAAGCTTGTCCTCCAA |
| *CsCHLG,bchG* | F: GTGCCGAAGCCAAAAGAAG R: GAAATGGGATGCGGGAAA |
| *CsRCA* | F: GGTCAGGGTAAATCTTTCCA R: CGGCATTTCCACTTTCCA |
| *Csrbcl* | F: TATGCGTTGGAGAGACCGTT R: CATGTACCCGCAGTAGCATTC |
| *CsPETD* | F: TCCTTTTGCAACTCCTTTGG R: CCGCTGGTACTGAAACCATT |
