# Supplementary material for: Light control of catechin accumulation is mediated by photosynthetic capacity in tea plant (Camellia sinensis)
Source: BMC Plant Biol. 2021 Oct 20;21:478. doi: 10.1186/s12870-021-03260-7 (PMC8527772; doi:10.1186/s12870-021-03260-7)
Supplement: Supplementary file 3 — Additional file 3: Supplementary Table 3. The cubic function equations in curve-fitting analysis. [file 12870_2021_3260_MOESM3_ESM.doc]

Supplementary table 3. The cubic function equations in [c](../../../../D:/Program%20Files%20(x86)/Youdao/Dict/8.9.3.0/resultui/html/index.html#/javascript:;)urve-fitting analysis

| X | Equations | R^2^ |
| --- | --- | --- |
| F | Y(EGCG)=11.148-0.096X+0.321X^2^+0.915X^3^ | 79.6% |
|  | Y(TNEC)=1.254+0.016X+0.325X^2^+0.227X^3^ | 47.9% |
|  | Y(TEC)=16.136-0.446X-0.268X^2^+1.091X^3^ | 75.8% |
|  | Y(TC)=17.390-0.430X+0.057X^2^+1.318X^3^ | 69.7% |
| F1 | Y(EGCG)=11.646+0.234X-0.594X^2^+0.012X^3^ | 72.4% |
|  | Y(TC)=18.093+0.036X-1.030X^2^+0.141X^3^ | 67.0% |
|  | Y(TNEC)=1.384+0.353X-0.051X^2^-0.251X^3^ | 74.3% |
|  | Y(TEC)=16.709-0.317X-0.979X^2^+0.392X^3^ | 65.9 |
