# Supplementary material for: Light control of catechin accumulation is mediated by photosynthetic capacity in tea plant (Camellia sinensis)
Source: BMC Plant Biol. 2021 Oct 20;21:478. doi: 10.1186/s12870-021-03260-7 (PMC8527772; doi:10.1186/s12870-021-03260-7)
Supplement: Supplementary file 4 — Additional file 4: Supplemental Figure 1. The PLS analysis between catechins content and the indexes of photosynthesis capacity of tea plants under different light intensity. [file 12870_2021_3260_MOESM4_ESM.doc]

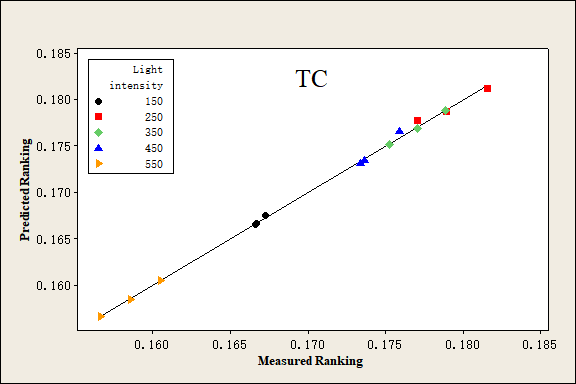

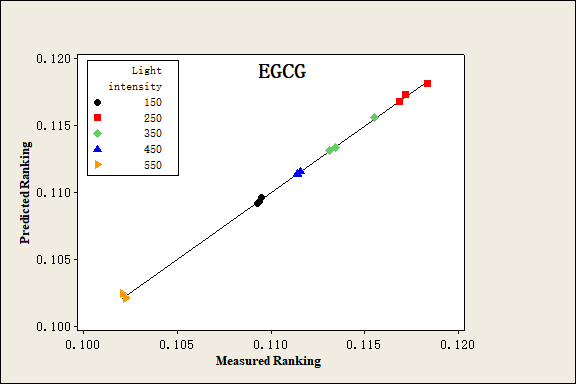

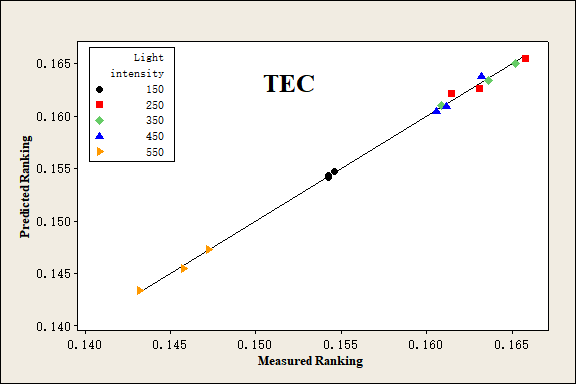

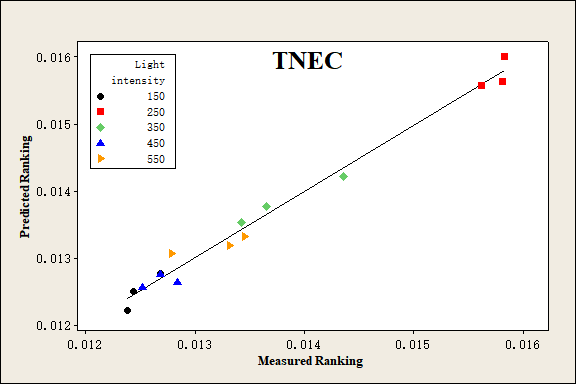


Supplemental figure1. The PLS analysis between catechins content and the indexes of photosynthesis capacity of tea plants under different light intensity.
