# Supplementary material for: Light control of catechin accumulation is mediated by photosynthetic capacity in tea plant (Camellia sinensis)
Source: BMC Plant Biol. 2021 Oct 20;21:478. doi: 10.1186/s12870-021-03260-7 (PMC8527772; doi:10.1186/s12870-021-03260-7)
Supplement: Supplementary file 5 — Additional file 5: Supplemental Figure 2. The expression of related genes under the different light intensity and their clustering. [file 12870_2021_3260_MOESM5_ESM.doc]

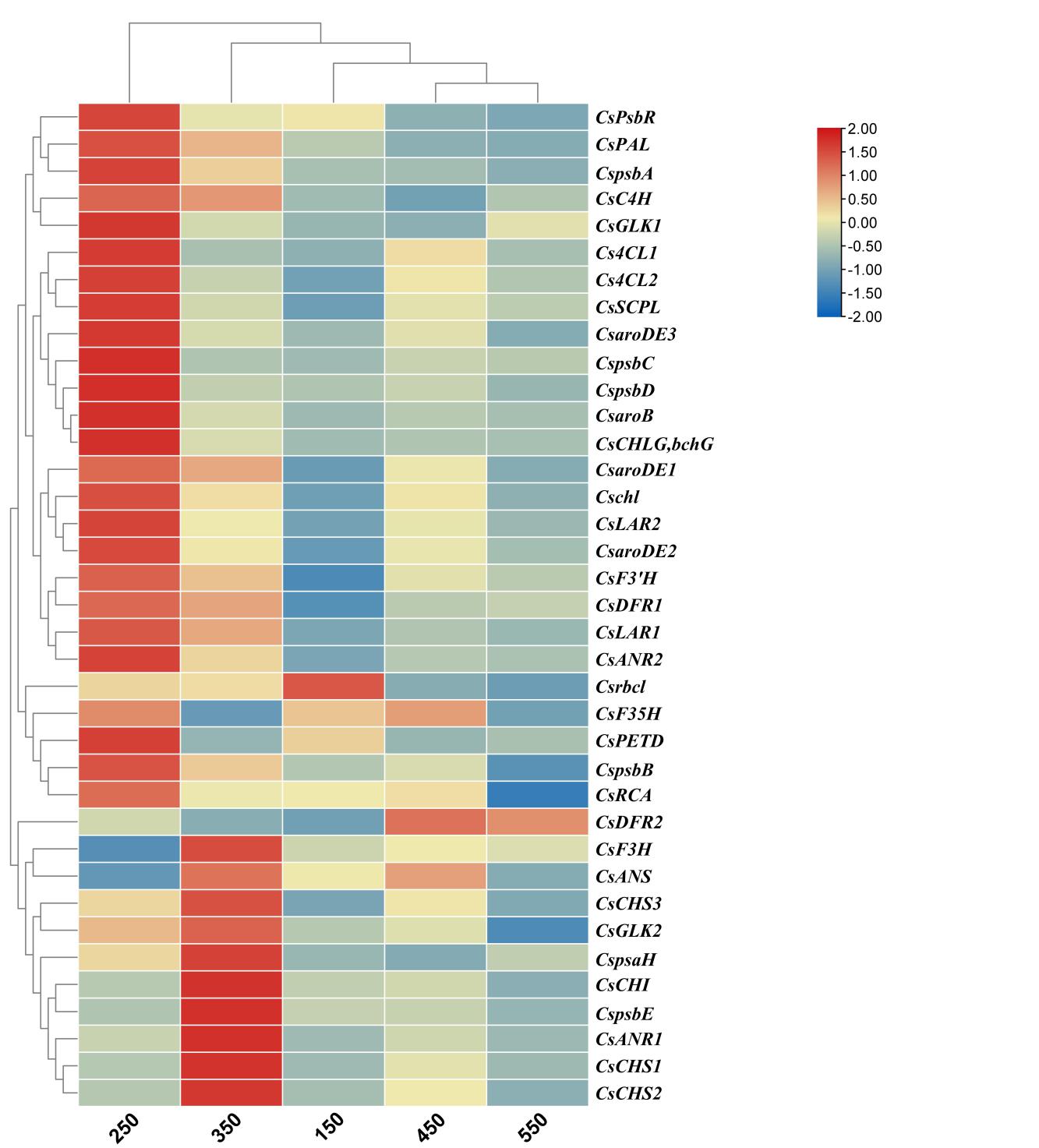


Supplemental figure 2. The expression of related genes under the different light intensity and their clustering
